# Retraction: Shah, A.M., et al. Glutamine Metabolism and Its Role in Immunity, a Comprehensive Review. *Animals* 2020, *10*, 326

**DOI:** 10.3390/ani11030905

**Published:** 2021-03-22

**Authors:** Animals Editorial Office

**Affiliations:** MDPI, St. Alban-Anlage 66, 4052 Basel, Switzerland

The journal retracts the article “Glutamine Metabolism and Its Role in Immunity, a Comprehensive Review” cited above [1].

Following publication, concerns were brought to the attention of the publisher regarding a high degree of similarity with a previously published article [2].

Adhering to our complaints procedure, an investigation was conducted that confirmed the extent of the overlap. The authors agreed to this retraction and would like to apologize for any inconvenience caused to the readers.

This retraction was approved by the Editor in Chief of the journal *Animals*.
